# On the theory of synchrotron radiation nano­focusing with planar compound refractive lenses

**DOI:** 10.1107/S1600577522001345

**Published:** 2022-03-14

**Authors:** V. G. Kohn

**Affiliations:** a National Research Centre ‘Kurchatov Institute’, 123182 Moscow, Russia; b Shubnikov Institute of Crystallography of Federal Scientific Research Centre ‘Crystallography and Photonics’ of Russian Academy of Sciences, 119333 Moscow, Russia

**Keywords:** X-ray nanofocusing, computer simulations, compound refractive lens, synchrotron radiation, theory of X-ray optics

## Abstract

Two new methods of computer simulation of synchrotron radiation nano­focusing with planar compound refractive lenses (PCRLs) are presented. The methods are based on the results of analytical theory. In contrast to previous works, the new methods take into account the PCRL aperture. It is especially important at high photon energies, when absorption is low and the calculations based on analytical theory, *i.e.* without taking into account the aperture, give incorrect results. A computer program was created and specific results were obtained for a silicon PCRL having an aperture of 50 µm, element length of 102 µm and minimum thickness of 2 µm. For an energy of 50 keV and number of elements 300, it focuses the beam to 31 nm size at a distance of one and a half times its length. Analysis of the calculation accuracy for the proposed methods is performed, as well as a demonstration of the capabilities of the computer program.

## Abstract 2

Two new methods of computer simulation of synchrotron radiation nanofocusing with planar compound refractive lenses (PCRLs) are presented. The methods are based on the results of analytical theory. A new feature is the possibility to take into account the PCRL aperture. A universal computer program was elaborated and specific results were obtained.

## 1. Introduction

Focusing of X-ray beams due to refractive effects was realized only a hundred years after the discovery of X-rays (Snigirev *et al.*, 1996 ▸). Today, planar compound refractive lenses (PCRLs) are one of the main X-ray optics tools at third- and fourth-generation synchrotron radiation (SR) sources and X-ray free-electron lasers. PCRLs make it possible to form beams in focus with nanometre transverse size. They are created on the surface of crystals (mainly silicon) by means of microstructuring methods and have a parabolic surface profile with very high precision, which is required for compressing the beam into such a small size.

**Figure 1:**
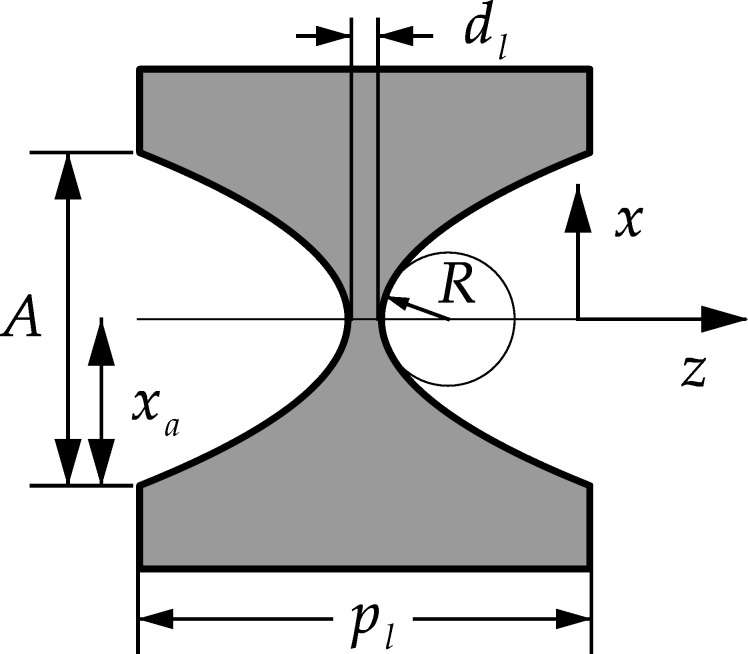
One element of the PCRL and its parameters.

Typical examples of PCRLs made of silicon are presented in works by Snigirev *et al.* (2009 ▸, 2014 ▸) and Zverev *et al.* (2021 ▸). They look like a sequence of elements in which each element is a biconcave lens, as shown in Fig. 1 ▸. A large number of elements is needed for two reasons. First, to eliminate aberrations it is necessary to gradually deflect the rays over the entire width of the beam inside the aperture. This is especially important for nanofocusing when the focal length is comparable with the length of the lens itself in the longitudinal direction. Second, it is very difficult to accurately manufacture a parabolic surface with a small radius of curvature and a long length along the optical axis. This shape is needed for X-ray lenses because the refraction of hard X-rays is very weak. It is easier to make large-radius lenses and use them many times in a sequence.

The theory of X-ray radiation propagation in such systems has features that are absent in the optics of visible light. The long PCRL propagator was first calculated analytically by Kohn (2002 ▸, 2003 ▸). Two approximations were made in this calculation for the correct solution of the problem. First, instead of discrete refraction at the boundaries of the surfaces of the PCRL elements, a medium with a parabolic profile of the electron density in the transverse direction and homogeneous in the longitudinal direction (the approximation of smeared lenses) was considered. Second, the real aperture of the PCRL was not taken into account, since it was assumed that the rays entering the PCRL at the boundaries of the aperture are completely absorbed.

Later, in works by Kohn (2009 ▸, 2012 ▸), another approach was developed, which also did not take into account the PCRL aperture, but used the compressed lens approximation. In this model, refraction and absorption took place in a thin transverse layer in the middle of the element length, and this is the same as for the full thickness of the element. There was no material between the layers. It was shown that in such a system a Gaussian beam retains its shape and only its complex parameters change. If a point source is deviated from the optical axis, then three complex parameters are required for a complete representation of the SR wavefunction. Recurrent equations were obtained. As a result, any set of elements can be easily computed numerically, using iterations to account for each subsequent element.

The compressed lens approximation allows one to correctly determine the effective aperture of the PCRL (Kohn, 2017 ▸), and to calculate the rocking curve FWHM (full width at half-maximum) and the coherence length for the long PCRL (Kohn, 2018*a* ▸). An online computer program was created (Kohn, 2018*b* ▸) to calculate all the specified parameters of the PCRL. At the same time, this approximation, as well as the previous approximation of smeared lenses, did not allow for correctly determining the nanofocusing limits using the PCRL, since for this purpose it was necessary to calculate the PCRL with weak absorption, where the PCRL aperture plays an important role.

The first attempt to take into account the aperture within the framework of the analytical theory was made by Kohn & Folomeshkin (2021 ▸), where it was shown that the minimum beam size at the focus can be obtained only at high photon energies, when absorption is minimal. Moreover, the smaller the aperture, the lower the energy that can be used for this purpose. However, the minimum beam size itself does not depend on the energy and aperture.

This work is a continuation and development of the analytical theory of focusing by means of PCRLs. It presents two new methods for a relatively accurate and fast numerical calculation of nanofocusing and gives a comparison with the previously known iterative method, as well as with analytical theory. It is shown that new calculation methods give beam intensity profiles at the focus with sufficient accuracy and lead to a reduction in the calculated time by a factor of 50 or more. The new calculation methods are implemented in the framework of a universal computer program, which is permanently under development with the aim of using it on SR sources and X-ray free-electron lasers to simulate all optical phenomena.

## 2. Methods of computer simulation of nanofocusing SR by means of PCRLs

SR is generated by electrons moving along a circular orbit at speeds close to the speed of light. Electrons create pulses of electromagnetic radiation with a very short duration and spontaneously. Different pulses are created at different time instants. We consider the case when a lot of pulses hit a detector during measurement. In such a case the experimental results are described successfully in an approximation where different points of the source cross section and different frequencies in the wide radiation spectrum shine incoherently; see, for example, the paper by Afanas’ev & Kon (1977 ▸). Therefore, it is necessary to solve the Maxwell wave equation for a point monochromatic source, and then to make a summation of the radiation intensity at the detector over the transverse size of the source and over the spectrum.

We assume that the experimental setup contains a monochromator, and the spectrum of radiation incident on the PCRL is very narrow. Therefore summation over the spectrum is not necessary. On the other hand, the monochromator does not influence the spatial properties of the X-ray beam, and we can omit it in our theory. Sometimes the summation over the transverse size of the source can be calculated as a convolution, but in general it can be a more difficult task. We assume as well that the beam width is greater than the PCRL aperture, and the finite angular divergence of SR does not influence the results.

As a first stage, it is necessary to consider the nanofocusing of X-ray radiation from a point monochromatic source. We note that such a source is absent in nature. It is only a theoretical tool with the aim to consider the coherence properties of SR. Let us choose the *z*-axis along the direction from the source to the centre of the PCRL, and the *x*-axis along the focusing direction. The PCRL does not influence the radiation along the *y*-axis. SR is linearly polarized and dipole scattering does not change its polarization. For this reason we are only interested in the modulus of the electric field, which at a large distance from the source can be represented as 

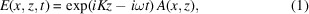

where *t* is the time, *K* = ω/*c* = 2π/λ is the wavenumber, ω is the circular frequency of radiation, *c* is the speed of light, and λ is the wavelength. The function *A*(*x*, *z*) changes slowly in space compared with the exponential. Let us call it the wavefunction (WF). We note that the WF depends on the frequency ω as well as on many other parameters, but we are interested in the WF spatial dependence.

Substitution of equation (1) into Maxwell’s wave equation for *E*(*x*, *z*, *t*) leads to a parabolic equation for the WF, 

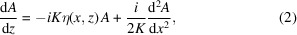

where η = δ − *i*β = 1 − *n*, *n* = ɛ^1/2^ is the complex refractive index of the medium taking into account absorption, and ɛ is the dielectric function. In equation (2) the second derivative over *z* is omitted in accordance with the paraxial approximation. It is negligible compared with the first derivative due to the large value of *K* for X-rays.

The standard calculation method for describing the propagation of SR along the optical axis is as follows. There are large distances in empty space when η = 0. In empty space, the solution to equation (2) with the boundary condition in the form of a delta function δ(*x*) is the Fresnel propagator, 

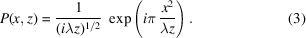

The solution of equation (3) is obtained by means of Fourier transformation. Then the general solution is obtained as a convolution, 

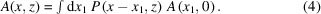

The convolution integral of equation (4) can be easily calculated by means of Fourier transformation. The standard way to do this is by use of the fast Fourier transform (FFT) numerical procedure proposed by Cooley & Tukey (1965 ▸).

### 2.1. The compressed lenses approach

In many cases objects have a relatively small thickness along the *z*-axis. For small distances the contribution of the second term in equation (2) can be neglected in comparison with the first term. In this approximation, the solution can be written as a product of two terms, 

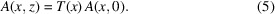

The function *T*(*x*) is called the transmission function. Consider homogeneous objects with complex shape. In this case, the optical properties of the object material do not depend on the coordinates; only the path length of the rays inside the object material is variable. In this case, 

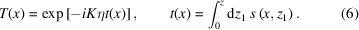

Here the parameter η is a constant and the function *s*(*x*, *z*_1_) equals 1 for the points where the material exists and 0 where it is absent.

For one PCRL element in the form of a biconcave parabolic lens (Fig. 1 ▸), it is easy to obtain the function *t*(*x*) in the form 

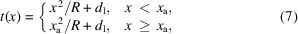

where *R* is the curvature radius at the apex of the parabola, *d*_l_ is the minimum thickness, and *x*_a_ = *A*/2 is half of the PCRL aperture. It is easy to understand that the parameter *p*_l_ = *t*(*x*_a_) is the length of one element of the PCRL along the *z*-axis. The parameters *x*_a_, *R*, *d*_l_ and *p*_l_ are shown in Fig. 1 ▸. The solution presented in equation (5) does not take into account the length of the object along the *z*-axis in any way. It is believed that the WF simply changes the phase as well as the amplitude in accordance with the absorption, which is described by the imaginary part of the phase.

The solution (7) is used in many works where the experimental method of phase contrast imaging (Snigirev *et al.*, 1995 ▸) is simulated. In such simulations the length of the objects is less than distances in empty space and can be neglected. However, in our case a more correct approximation is necessary where the lens adds a phase factor according to equation (5) at its length centre, while before and after this centre the radiation passes through empty space at a distance of *p*_l_/2. These distances can be taken into account by use of equation (4). For a PCRL with *n*_l_ elements, first we must take into account the distance *p*_l_/2, then (*n*_l_ − 1) times the combination of the function *T*(*x*) and the distance *p*_l_, then again the function *T*(*x*) and the distance *p*_l_/2.

This method of calculation can be called the compressed lenses approach (CLA). Further, it is referenced as the first method. If the number of elements *n*_l_ is large, the calculation takes a relatively long time. Its advantage is that it is able to take into account the aperture of the PCRL, as well as several PCRLs in the transverse direction, that is, the bi-lens and multi-lens interferometer. In addition, it allows one to propagate an arbitrary WF.

### 2.2. The smeared lenses approach

The second method uses an analytical long PCRL propagator, considered by Kohn (2002 ▸, 2003 ▸). It allows one to significantly reduce the time for calculating the PCRL, which consists of a large number of elements. In work by Kohn (2003 ▸), an image propagator was obtained using the PCRL at any distance before and after the lens. But the difficulty is that this propagator does not take into account the lens aperture. For this reason, we are not interested in the distance in front of the PCRL and we need to consider the case of zero distance. In this case, the aperture can be taken into account by installing a slit in front of the PCRL with a size equal to the size of the PCRL aperture. This, indeed, is often done in experiments. The distance after the slit *z* can be left arbitrary.

This approach leads to the following general solution, 

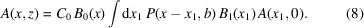

Here, *P*(*x*, *b*) is the Fresnel propagator for the complex distance *b*, 

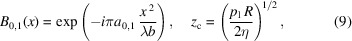




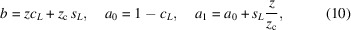




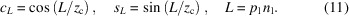

It is important to remember that here the distance *z* is measured from the end of the PCRL, and the function *A*(*x*_1_, 0) corresponds to the beginning of the PCRL. The length *L* of the PCRL is taken into account explicitly. The multiplier *C*_0_ = 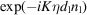 does not affect focusing. It simply reduces the intensity of the beam by absorption in the flat area of the material, which is necessary for technical reasons.

It should be noted that in the derivation of the long PCRL propagator the opposite approximation of smeared lenses was used. Namely, it was assumed that the function η(*x*, *z*) in equation (2) is independent of *z* and has the form 

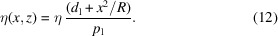

Here, the parameter η on the right-hand side is again a constant, and at each point along the *x*-axis it is taken with a weight equal to the ratio of the thickness of the material in the lens to the total thickness of the lens, that is, at *x* = *x*_a_. However, for *x* > *x*_a_ the equation remains the same, that is, incorrect. It is believed that the WF there is already equal to zero. If this is not the case, then the solution is incorrect and cannot be used. That is why it is necessary to confine the beam with a slit in front of the PCRL having a width equal in size to the aperture of the PCRL, namely, 2*x*_a_. This second method we will call the smeared lenses approach (SLA).

The presence of two methods for solving the problem in directly opposite approximations makes it easy to assess the accuracy of the results obtained. It will be determined by the difference in solutions obtained in two ways. If they match, we can conclude that both methods give the correct solution.

In equation (8), the integral has the form of a convolution of two functions *P*(…) and *B*_1_(…)*A*(…) and it can be calculated by the FFT method. In this case, the WF is first multiplied by *B*_1_(*x*). Then the convolution is calculated. Finally, one has to multiply again the result by *C*_0_ *B*_0_(*x*). Such a calculation needs to be performed only once for any number *n*_l_ of PCRL elements.

The second method has an alternative when the WF is calculated at *z* = 0, *i.e.* at the end of the PCRL. In such a variant the distance *z* is taken into account by formula (4). Practice has shown that this option is more resistant to the accumulation of errors in using the FFT method, and it works correctly with a wider choice of parameters of the computational grid. In this case, the increase in the calculation time turns out to be small. The second method should be used if a large number of calculations for various parameter values are required.

### 2.3. The approximate smeared lenses approach

It is useful to consider a third method. It is approximate, but even faster than the second method. The formula (8) at *z* = 0 can be rewritten as a matrix multiplication of the PCRL propagator by the input WF,

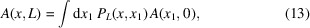

where 

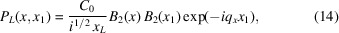




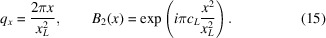

Here, *x*_*L*_ = (λ*z*_c_ *s*_*L*_)^1/2^, and the parameters *z*_c_, *s*_*L*_ and *c*_*L*_ are defined above.

It is easy to see that the integral is the Fourier transform. But the wavevector *q*_*x*_ is complex and it is impossible to calculate the integral by standard methods. However, one can obtain an approximate value of the integral by means of the stationary phase method (Jeffreys & Swirles, 1966 ▸) assuming that the input WF is slowly dependent on its argument. In the integral (13), the stationary phase point *x*_0_ = *x*/*c*_*L*_. We replace the function *A*(*x*_1_, 0) by the constant *A*(*x*_0_, 0) after which the integral is calculated exactly and the result is obtained in the analytical form 

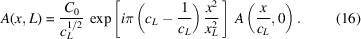

Obviously, when a plane wave is incident along the optical axis, this result is accurate. But this case does not suit us, since the input WF should be limited by the slit. In the numerical calculation, the imaginary part of the parameter *c*_*L*_ can be neglected everywhere, except for the exponential. The real part of *c*_*L*_ can be calculated as 

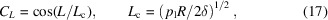

for the case with β = 0.

Interestingly, a physically important conclusion immediately follows from equation (16). If absorption is absent or weakly affects the exponential, then the input beam, limited by the slit in front of the PCRL, is compressed at the end of the PCRL by a factor of *C*_*L*_. This fully corresponds to the trajectory of the ray inside the PCRL, which starts at the edge of the aperture. The ray trajectories were discussed by Kohn & Folomeshkin (2021 ▸). This third method we will call the approximate smeared lenses approach (ASLA).

## 3. Computer simulations and their analysis

The three calculation methods (CLA, SLA and ASLA) considered in the preceding section were implemented in the computer program *XRWP1* 1, which is permanently being developed to simulate the propagation of a SR beam through all elements of the optical scheme at stations of third- and fourth-generation SR sources and X-ray free-electron lasers. The program is written in the special command language ACL (Kohn, 2021 ▸), which is executed by an interpreter program written in the Java programming language.

One of the first nanofocusing PCRLs made of silicon (Snigirev *et al.*, 2009 ▸) had the following parameters for the biconcave elements: aperture *A* = 50 µm, curvature radius *R* = *A*/8, minimum thickness *d*_l_ = 2 µm, length of one element *p*_l_ = *d*_l_ + *A* ^2^/4*R*. Later, PCRLs with apertures of 30 µm (Snigirev *et al.*, 2014 ▸) and 10 µm (Zverev *et al.*, 2021 ▸) were manufactured with the same ratios for the other parameters as indicated above.

As shown by Kohn & Folomeshkin (2021 ▸), all three types of PCRLs reach the size limit for nanofocusing, equal approximately to *w*_c_ = λ/(8δ)^1/2^, which depends only on the optical properties of the material. This means that the transverse size (FWHM) of the SR beam at the focus cannot be smaller. This estimate was first proposed by Bergemann *et al.* (2003 ▸) for all focusing devices of X-ray optics. For PCRLs, it is fulfilled only at high photon energies *E* when absorption does not play a significant role. Moreover, the larger the aperture, the higher the energy required for this.

Obviously, the minimum size of the SR beam is reached at the end of the PCRL. We will consider such a PCRL as having the maximum possible length. However, the second half of the PCRL length works less efficiently, and it is sufficient to consider the PCRL with a length approximately half of the maximum size. It is much more difficult to obtain an analytical estimate for the depth of focus; that is, the distance along the *z*-axis at which the minimum beam size is approximately maintained. The easiest way to estimate this is by direct computer simulation.

**Figure 2:**
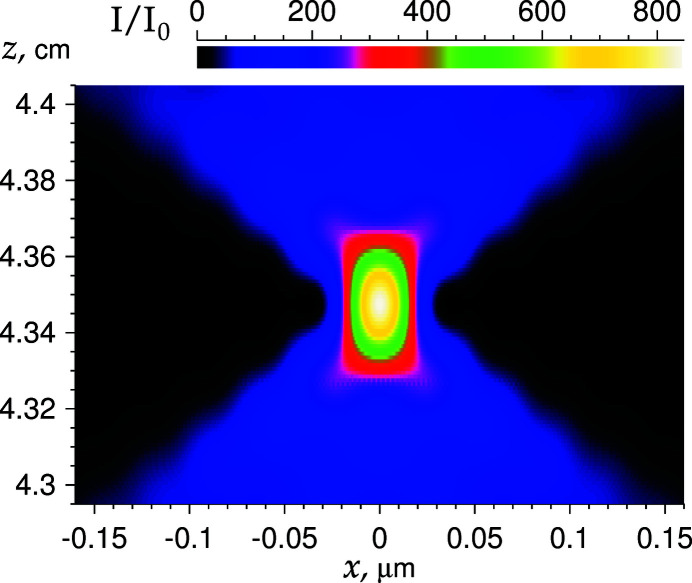
Relative intensity distribution near the focal distance. See details in the text.

Fig. 2 ▸ shows the distribution of the relative intensity of the SR beam on the (*x*, *z*) plane near the focus for a PCRL with an aperture of 50 µm and for the number of elements *n*_l_ = 300 at *E* = 50 keV. The calculation was performed using an alternative version of the second method described in the previous section. The distance from the point source to the beginning of the PCRL is *z*_0_ = 50 m. The convolutions were calculated using the FFT numerical procedure (Cooley & Tukey, 1965 ▸). The set of points with a constant step *d* = 0.00125 µm and number of points *N* = 2^16^ = 65536 was used. Such a large number of points is necessary because the size *Nd* of the region has to be larger than the aperture, and the narrow beam at the focus has to be shown with a relatively high resolution.

Intensity *I*_0_ corresponds to the intensity at the entrance face of the PCRL, in front of which there is a slit. The slit size is equal to the size of the aperture. It follows from the figure that the focal length, measured from the end of the PCRL, is 4.347 cm. This value is the same as that calculated by the online calculator developed by Kohn (2018*b* ▸). On the other hand, this distance is slightly larger than the PCRL length *L* = 3.06 cm. The distance can be used to insert a PCRL with a smaller aperture, which will focus the beam in the *y* direction.

Let us define the depth of focus (DF) as the width at half the height of the intensity distribution curve along the optical axis. The calculation shows that the DF can be estimated as 0.3 mm (the height of the green region in Fig. 2 ▸), which is 10^4^ times larger than the transverse size of the beam, equal to 31 nm.

**Figure 3:**
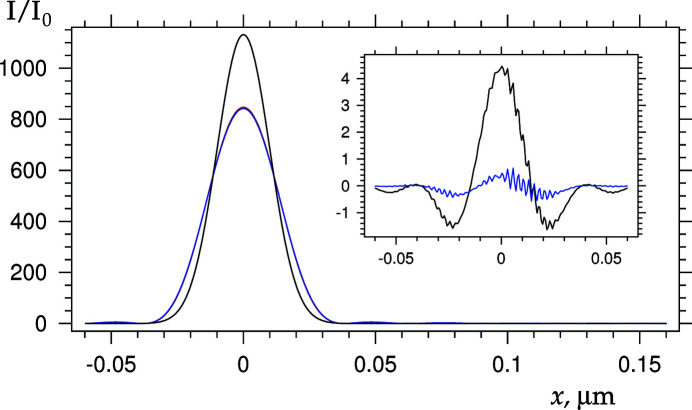
Relative intensity distribution at the focal distance. See details in the text.

Fig. 3 ▸ shows the relative intensity distribution at a focal length of 4.347 cm, calculated using four different methods. Three of the methods (CLA, SLA and ASLA) are described in the previous section; the fourth one makes use of equations (17) and (18) of Kohn (2003 ▸), taking into account the distances before and after the PCRL, but without taking into account the aperture. Let us indicate the curves by the number of the calculation method. Fig. 3 ▸ presents the first curve (CLA method) as black, the second one (SLA method) as red, the third (ASLA method) as blue, and the fourth as black again.

The fourth curve is, by definition, a Gaussian function with a higher maximum and lower FWHM. The first three curves practically coincide. In this case, only the blue curve is visible, which is drawn last. The black and red curves are very slightly different from the blue curve. The effect of the aperture can be judged by the difference in the shape of the blue and black curves. The effect of the aperture leads to a decrease in the maximum, an increase in FWHM, and the presence of weak additional maxima outside the main maximum.

As shown by Kohn & Folomeshkin (2021 ▸), the fourth curve is not correct because the parameters do not satisfy the condition *A*_e_ < *A*/2 when the effect of the aperture can be neglected. Here *A*_e_ is the effective aperture of the PCRL due to absorption. The online program for the parameters of the considered calculation gives the value *A*_e_ = 30 µm, which is greater than 25 µm. But the fourth curve does not differ much from the results of the more accurate calculation because the difference of *A*_e_ from *A*/2 is small.

The difference between the second and the first curves gives an estimate of the accuracy for both calculation methods, since the calculations were performed in directly opposite approximations of smeared and compressed PCRL elements. This difference is shown by the black curve in the inset of Fig. 3 ▸. The slight lack of symmetry and small oscillations in the curve are due to diffraction at the slit and the fact that the data are obtained by linear interpolation from a large data set with a small step.

The smeared lens method (second curve) gave a higher maximum in Fig. 3 ▸, but lower values on the tails. However, the relative difference is 0.005 at the maximum. The second curve is also calculated 50 times faster than the first curve. The difference between the second and third curves is shown by the blue curve in the inset; this difference is even smaller. The reason for this is that the source is located at a large distance of 50 m in the case under consideration, and the incident wave is close to a plane wave. For a plane wave, the third method gives an accurate result.

It is interesting to compare the results of the accurate calculation by means of program *XRWP1* with the known results of the approximate analytical theory for the focus distance. It is clear that with a small *L*/*F* ratio a long lens can be roughly considered as a short one, placed in its middle. Here *F* = *R*/2*n*_l_δ is the focal length for a short lens, which does not take into account its length *L*. It was shown by Kohn and co-workers (Kohn *et al.*, 2003 ▸; Kohn, 2003 ▸) that a more accurate focal length for a parallel beam will be equal to *F*_*L*_ = *F* + *L*/6. This result takes into account only the first term of the expansion in the small parameter *L*/*F*. For the computer simulation, we used the parameters *F* = 5.4 cm, *F*_*L*_ = 5.91 cm.

The accurate result for the focal length measured from the middle of the PCRL length and converted to a parallel-beam case is *F*_a_ = 5.87 cm. The approximate estimate for the focal length exceeds the accurate calculation, but the difference is small, although the ratio *L*/*F* = 0.57 is not so small. But the difference is still greater than the depth of focusing.

Up to now we have considered a point source. However, a real source of SR has a transverse size. It contains many point sources distributed along the *x*-axis. As shown by Kohn (2009 ▸, 2012 ▸), when the point source is displaced and has the coordinate *x*_*s*_, the beam, focused by the long PCRL, will be displaced without distortion in the opposite direction at the distance *Mx*_*s*_, and the magnification factor *M* = (*z* + *Z*_1_)/(*z*_0_ + *Z*_0_), where the distance *z* is counted from the end of the PCRL to the detector. The parameters *Z*_0_ and *Z*_1_ are calculated by the online program (Kohn, 2018*b* ▸). So, for the PCRL under consideration, we obtain *Z*_0_ = 77 cm, *Z*_1_ = 1.7 cm. Since *z*_0_ = 5000 cm, the influence of the parameter *Z*_0_ is small, and *Z*_1_ > *L*/2 = 1.53 cm. The results of accurate calculation correspond to the *M* values obtained from the online program, at least at relatively small point source displacements, of no more than 100 µm.

**Figure 4:**
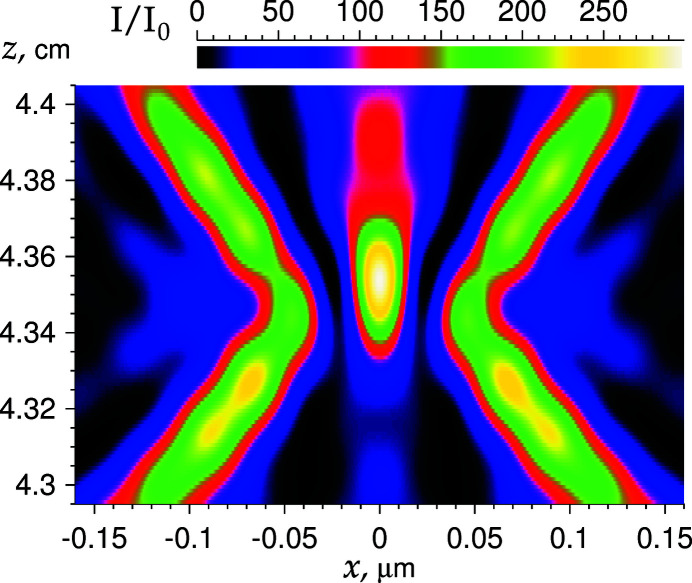
Relative intensity distribution near the focal distance for the parameters as in Fig. 2 ▸, but with a tungsten wire in front of the PCRL. See details in the text.

We have considered a simple example of calculating the focusing of an X-ray beam using a nanofocusing PCRL only for the purpose of demonstrating the operation of the program. The program is able to perform calculations for more complex experimental schemes, in which real objects are depicted, an interference pattern is formed, a PCRL cascade with various apertures is used, and so on. Fig. 4 ▸ shows an example of a more complex system, where a tungsten wire of diameter 10 µm is located at zero distance in front of the slit and the PCRL.

The calculation was performed by the same method as used for Fig. 2 ▸. It should be noted that in calculations using the FFT procedure the correct choice of calculation parameters, namely the step and the number of points, is very important. If the parameters are chosen incorrectly, then the result may contain artefacts in the form of oscillations with a small period, or may be completely wrong with even abnormally large numbers, many orders of magnitude larger than the real ones. In particular, in order to obtain the correct result of calculation in Fig. 4 ▸, it was necessary to increase the grid step twice, *i.e.* *d* = 0.0025 µm. Calculation by the third method in this case gave the same result as well.

## 4. Conclusion

Nanofocusing planar compound refractive lenses consist of a large number of elements and an accurate calculation of their focusing properties takes a lot of time. The analytical theory developed by Kohn (2002 ▸, 2003 ▸) is inapplicable for such lenses, because it does not take into account the size of the aperture. In this work, two new methods for the computer simulation of nanofocusing planar compound refractive lenses are developed. They are based on the analytical theory, but with careful consideration of the aperture size. New methods make it possible to reduce the calculation time by 50 or more times without loss of accuracy. An analysis of the accuracy of the new methods is carried out on a specific example of nanofocusing lenses already used in experiments. The new methods were implemented in a universal computer program.

## Footnotes

1 The program *XRWP1* is not ready for free download. In case of interest, please email the author (kohnvict@yandex.ru).
